# The potential use of the *Penicillium chrysogenum* antifungal protein PAF, the designed variant PAF^opt^ and its γ‐core peptide Pγ^opt^ in plant protection

**DOI:** 10.1111/1751-7915.13559

**Published:** 2020-03-24

**Authors:** Liliána Tóth, Éva Boros, Péter Poór, Attila Ördög, Zoltán Kele, Györgyi Váradi, Jeanett Holzknecht, Doris Bratschun‐Khan, István Nagy, Gábor K. Tóth, Gábor Rákhely, Florentine Marx, László Galgóczy

**Affiliations:** ^1^ Institute of Plant Biology Biological Research Centre Temesvári krt. 62 H‐6726 Szeged Hungary; ^2^ Institute of Biochemistry Biological Research Centre Temesvári krt. 62 H‐6726 Szeged Hungary; ^3^ Department of Plant Biology Faculty of Science and Informatics University of Szeged Közép fasor 52 H‐6726 Szeged Hungary; ^4^ Department of Medical Chemistry Faculty of Medicine University of Szeged Dóm tér 8 H‐6720 Szeged Hungary; ^5^ Institute of Molecular Biology Biocenter Medical University of Innsbruck Innrain 80‐82 A‐6020 Innsbruck Austria; ^6^ MTA‐SZTE Biomimetic Systems Research Group University of Szeged Dóm tér 8 H‐6720 Szeged Hungary; ^7^ Department of Biotechnology Faculty of Science and Informatics University of Szeged Közép fasor 52 H‐6726 Szeged Hungary; ^8^ Institute of Biophysics Biological Research Centre Temesvári krt. 62 H‐6726 Szeged Hungary

## Abstract

The prevention of enormous crop losses caused by pesticide‐resistant fungi is a serious challenge in agriculture. Application of alternative fungicides, such as antifungal proteins and peptides, provides a promising basis to overcome this problem; however, their direct use in fields suffers limitations, such as high cost of production, low stability, narrow antifungal spectrum and toxicity on plant or mammalian cells. Recently, we demonstrated that a *Penicillium chrysogenum*‐based expression system provides a feasible tool for economic production of *P. chrysogenum* antifungal protein (PAF) and a rational designed variant (PAF^opt^), in which the evolutionary conserved γ‐core motif was modified to increase antifungal activity. In the present study, we report for the first time that γ‐core modulation influences the antifungal spectrum and efficacy of PAF against important plant pathogenic ascomycetes, and the synthetic γ‐core peptide Pγ^opt^, a derivative of PAF^opt^, is antifungal active against these pathogens *in vitro*. Finally, we proved the protective potential of PAF against *Botrytis cinerea* infection in tomato plant leaves. The lack of any toxic effects on mammalian cells and plant seedlings, as well as the high tolerance to harsh environmental conditions and proteolytic degradation further strengthen our concept for applicability of these proteins and peptide in agriculture.

## Graphical abstract

The emerging number of crop losses due to infection or contamination caused by pesticide‐resistant pre‐ and post‐harvest plant pathogenic fungi urges the need for the development of fundamentally new and safe antifungal strategies in the agriculture, and the cysteine‐rich, highly stable antifungal peptides and proteins from filamentous ascomycetes are promising candidates in this respect. We report for the first time that γ‐core modulation influences the antifungal spectrum and efficacy of *Penicillium chrysogenum* antifungal protein (PAF) against important plant pathogenic ascomycetes, and the synthetic γ‐core peptide Pγ^opt^, a derivative of an engineered variant of PAF is antifungal active against these pathogens. We also prove the protective potential of PAF against *Botrytis cinerea* infection in tomato plant leaves, and the potential applicability of PAF, and its engineered variant as biofungicides in the agriculture as they do not show any toxic effects on mammalian cells and plant seedlings, and they have high tolerance to harsh environmental conditions and proteolytic degradation.

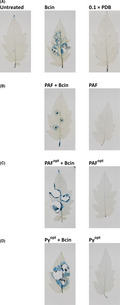

## Introduction

The incidence of infectious diseases caused by plant pathogenic fungi shows an increasing trend worldwide in the last years and causes enormous crop losses in agriculture (Fisher *et al.*, 2012). The reason for this phenomenon is multifactorial. The genome structure of fungal pathogens can affect the evolution of virulence (Howlett *et al.*, 2015), and uniform host population can facilitate the pathogen specialization and speciation to overcome plant resistance genes and applied pesticides (McDonald and Stukenbrock, 2016). The climate change (Elad and Pertot, 2014), global trade and transport (Jeger *et al.*, 2011) promote dispersal and invasion of plant pathogenic fungi in agroecosystems. Furthermore, invasive weeds are able to facilitate emergence and amplification of recently described or undescribed new fungal pathogens in agricultural fields (Stricker *et al.*, 2016). Another important aspect is the potential for plant pathogenic fungi to rapidly evolve resistance mechanisms against licensed and widely used fungicides (Lucas *et al.*, 2015), which is common on farms all over the world (Borel, 2017). Fungi can adapt to them by *de novo* mutation or selection from standing genetic variation (Hawkins *et al.*, 2019) leading to resistance and loss of fungicide efficacy (Hahn, 2014). This problem is further exacerbated by the application of analogues of antifungal drugs (such as azoles) in agriculture resulting in parallel evolution of resistance mechanism in the clinic and the fields (Fisher *et al.*, 2018), and in the global spread of resistant genotypes (Wang *et al.*, 2018). Recently, more than two hundred agriculturally important fungal species have been registered as resistant to at least one synthetic pesticide in the CropLife International database (Borel, 2017). In order to resolve the problem of emerging and accelerated resistance development of plant pathogenic fungi (Lucas *et al.*, 2015), new fungicides with different modes of action than the currently applied ones need to be discovered and introduced in agriculture to prevent a collapse in the treatment of fungal infections. However, the discovery of new fungicides has been very modest in the last years, because candidate drugs need to be highly fungal pathogen‐specific and producible at low costs. The high expenses to introduce new pesticides to the market, and the ability of fungi for fast resistance development further hamper the market accessibility of new compounds. Alternatives, such as microbes, genetic engineering and biomolecules provide a feasible solution to replace synthetic fungicides and thus to overcome resistance (Lamberth *et al.*, 2013; Borel, 2017).

Bacteria are already well‐known as a rich source of new fungicides as they are able to produce numerous antifungal compounds. The features of these secondary metabolites can fit to the recent requirements for agricultural disease control agents. These are the biodegradability, selective mode of action without exerting toxic effects on non‐fungal organisms, and low risk for resistance development (Kim and Hwang, 2007). They directly interfere with the fungal pathogen and mostly affect the integrity of cell envelope (e.g. several antifungal cyclic peptides; Lee and Kim, 2015), inhibit the fungal growth (e.g. 4‐hydroxybenzaldehyde; Liu *et al.*, 2020), or sexual mating (e.g. indole‐3‐carbaldehyde; Liu *et al.*, 2020). In spite of these advantages only few of them have been successfully developed into commercial fungicides. Beside these directly interfering compounds, bacteria in the rhizobiome produce different types of molecules that modulate the biosynthesis of plant‐derived natural products active against fungal plant pathogens (Thomashow *et al.*, 2019).

In contrast to the wide range of bacterial fungicides, little information can be found in the literature about biofungicide potential of secondary metabolites from fungal origin (Masi *et al.*, 2018). In the last two decades, several studies already demonstrated that the small, cysteine‐rich and cationic antifungal proteins secreted by filamentous ascomycetes (APs) could be considered as potential fungicides in agriculture (Leiter *et al.*, 2017) as they efficiently inhibit the growth of plant pathogenic fungi and protect the plants against fungal infections without showing toxic effects (Vila *et al.*, 2001; Moreno *et al.*, 2003, 2006; Theis *et al.*, 2005; Barna *et al.*, 2008; Garrigues *et al* *.*, 2018; Shi *et al* *.*, 2019). Transgenic wheat (Oldach *et al.*, 2001), rice (Coca *et al.*, 2004; Moreno *et al.*, 2005) and pearl millet (Girgi *et al.*, 2006) plants expressing the *Aspergillus giganteus* antifungal protein (AFP) have been bred, and they show less susceptibility to the potential fungal plant pathogens. However, the non‐coherent regulations for cultivation of genetically modified (GM) plants (Tagliabue, 2017), the limited acceptance of GM products by consumers, and the spreading anti‐GM organism attitude contradict their introduction in the agriculture (Lucht, 2015). Therefore, the traditional pest control, *viz.* the environmental application of chemicals in fields is still preferred (Bardin *et al.*, 2015).

In spite of the above discussed promising results, the narrow and species‐specific antifungal spectrum of APs limits their application as effective fungicides (Galgóczy *et al.*, 2010). Rational protein design based on their evolutionary conserved γ‐core motif (GXC‐X_[3‐9]_‐C) provides a feasible tool to improve the efficacy (Sonderegger *et al.*, 2018). The γ‐core motif can be found in pro‐ and eukaryotic, cysteine‐rich antimicrobial peptides and proteins (Yount and Yeaman, 2006) and plays an important role in the antifungal action of plant defensins (Sagaram *et al.*, 2011). In our previous study, we applied rational design to change the primary structure of the γ‐core motif in *Penicillium chrysogenum* antifungal protein (PAF) and to create a new PAF variant, PAF^opt^, with improved efficacy against the opportunistic human yeast pathogen *Candida albicans.* The improvement of the antifungal activity was achieved by the substitution of defined amino acids in the γ‐core motif of PAF to elevate the positive net charge and the hydrophilicity of the protein (Table 1). Electronic circular dichroism (ECD) spectroscopy indicated that these amino acid substitutions do not significantly affect the secondary structure and the β‐pleated conformation (Sonderegger *et al.*, 2018). Furthermore, the antifungal efficacy of two synthetic 14‐mer peptides, Pγ and Pγ^opt^ (Table 1), that span the γ‐core motif of PAF and PAF^opt^, respectively, was proven and higher anti‐*Candida* efficacy of Pγ^opt^ was reported (Sonderegger *et al.*, 2018).

**Table 1:** Amino acid sequence and *in silico* predicted physicochemical properties of PAF, PAF^opt^, Pγ and Pγ^opt^ according to Sonderegger *et al. *(2018).

Protein	Number of amino acids	Molecular weight (kDa)	Number of Cys	Number of Lys/Arg/His	Theoretical pI	Estimated charge at pH = 7.0	GRAVY
AKYT**GKCTKSKNECK**YKNDAGKDTFIKCPKFDNKKCTKDNNKCTVDTYNNAVDCD							
PAF	55	6.3	6	13/0/0	8.93	+4.7	_−_1.375
Ac‐KYTGKC(‐SH)TKSKNEC(‐SH)K‐NH_2_							
Pγ	14	1.6	2	5/0/0	9.51	+3.8	_−_1.814
AKYT**GKCKTKKNKCK**YKNDAGKDTFIKCPKFDNKKCTKDNNKCTVDTYNNAVDCD							
PAF^opt^	55	6.3	6	15/0/0	9.30	+7.7	_−_1.438
Ac‐KYTGKC(‐SH)KTKKNKC(‐SH)K‐NH_2_							
Pγ^opt^	14	1.7	2	7/0/0	10.04	+6.8	_−_2.064

GRAVY, grand average of hydropathy value. The γ‐core motif in the primary structure of the protein is indicated in bold and underlined letters.

The present study aimed at investigating the applicability of PAF^opt^ and the two synthetic γ‐core peptides Pγ and Pγ^opt^ as sole biocontrol agents in plant protection by comparing their antifungal spectrum against plant pathogenic filamentous fungi and the toxicity against different human cell lines and plant seedling with that of the wild‐type PAF. Furthermore, the potential application of PAF, PAF^opt^ and Pγ^opt^ as protective agents against fungal infection of tomato plant leaves was evaluated.

## Results

### In vitro susceptibility of plant pathogenic fungi to the P. chrysogenum APs

Broth microdilution susceptibility tests were performed to investigate the differences in the antifungal potency and spectrum of PAF, PAF^opt^ and the two synthetic γ‐core peptides Pγ and Pγ^opt^ against plant pathogenic fungi. The detected *in vitro* minimal inhibitory concentrations (MICs) against species belonging to genera *Aspergillus*, *Botrytis*, *Cladosporium* and *Fusarium* are summarized in the Table 2. PAF inhibited the growth of all isolates, except for *Fusarium boothi* and *Fusarium graminearum*, in the applied concentration range showing different MICs (from 1.56 µg ml^−1^ to 400 µg ml^−1^). In contrast, PAF^opt^ was ineffective against aspergilli, while *Cladosporium* and *Fusarium* isolates proved to be more susceptible to this PAF variant than to the native PAF, with exception of *Botrytis cinerea* SZMC 21472 and *Fusarium oxysporum* SZMC 6237J. This latter isolate showed the so‐called paradoxical effect, namely the fungus resumed growth at concentrations above the MIC (Table S1). While the synthetic γ‐core peptide Pγ was ineffective at concentrations up to 400 µg ml^−1^ (data not shown), its optimized variant Pγ^opt^ inhibited the growth of *B. cinerea* SZMC 21472 (MIC = 25 µg ml^−1^), *Cladosporium herbarum* (MIC = 12.5 µg ml^−1^) and all tested fusaria (MIC = 12.5–25 µg ml^−1^). These results indicated that the γ‐core modulation of PAF influences the antifungal spectrum and efficacy of the protein. Based on the results of these *in vitro* susceptibility tests, the antifungal effective proteins PAF and PAF^opt^, and the synthetic γ‐core peptide Pγ^opt^ were selected for further experiments.

**Table 2:** Minimal inhibitory concentrations (µg ml^−1^) of PAF, PAF^opt^ and Pγ^opt^ against plant pathogenic filamentous ascomycetes.

Isolate	PAF	PAF^opt^	Pγ^opt^	Origin of isolate
*Aspergillus flavus* SZMC 3014	3.125	> 400	> 400	*Triticum aestivum*/Hungary
*Aspergillus flavus* SZMC 12618	3.125	> 400	> 400	*Triticum aestivum*/Hungary
*Aspergillus nige*r SZMC 0145	3.125	> 400	> 400	Fruits/Hungary
*Aspergillus niger* SZMC 2759	3.125	> 400	> 400	Raisin/Hungary
*Aspergillus welwitschiae* SZMC 21821	1.56	> 400	> 400	*Allium cepa*/Hungary
*Aspergillus welwitschiae* SZMC 21832	1.56	> 400	> 400	*Allium cepa*/Hungary
*Botrytis cinerea* SZMC 21472	1.56	12.5	25	*Rubus idaeus*/Hungary
*Cladosporium herbarum* FSU 1148	100	12.5	6.25	n.d.
*Cladosporium herbarum* FSU 969	100	12.5	6.25	n.d
*Fusarium boothi* CBS 110250	> 400	200	12.5	*Zea mays*/South Africa
*Fusarium graminearum* SZMC 6236J	> 400	200	12.5	Vegetables/Hungary
*Fusarium oxysporum* SZMC 6237J	400	100^a^	25	Vegetables/Hungary
*Fusarium solani* CBS 115659	200	50	12.5	*Solanum tuberosum*/Germany
*Fusarium solani* CBS 119996	200	50	12.5	*Daucus carota*/The Netherlands

CBS, Centraalbureau voor Schimmelcultures, Utrecht, The Netherlands; FSU, Fungal Reference Centre University of Jena, Jena, Germany; SZMC, Szeged Microbiological Collection, University of Szeged, Szeged, Hungary. n.d., data not available.

^a^ Paradoxical effect was detected. *F. oxysporum* SZMC 6237J continued to grow in concentrations above the MIC.

### In vitro cytotoxic activity of PAF, PAF^opt^ and Pγ^opt^ on human cells

**Fig. 1:**
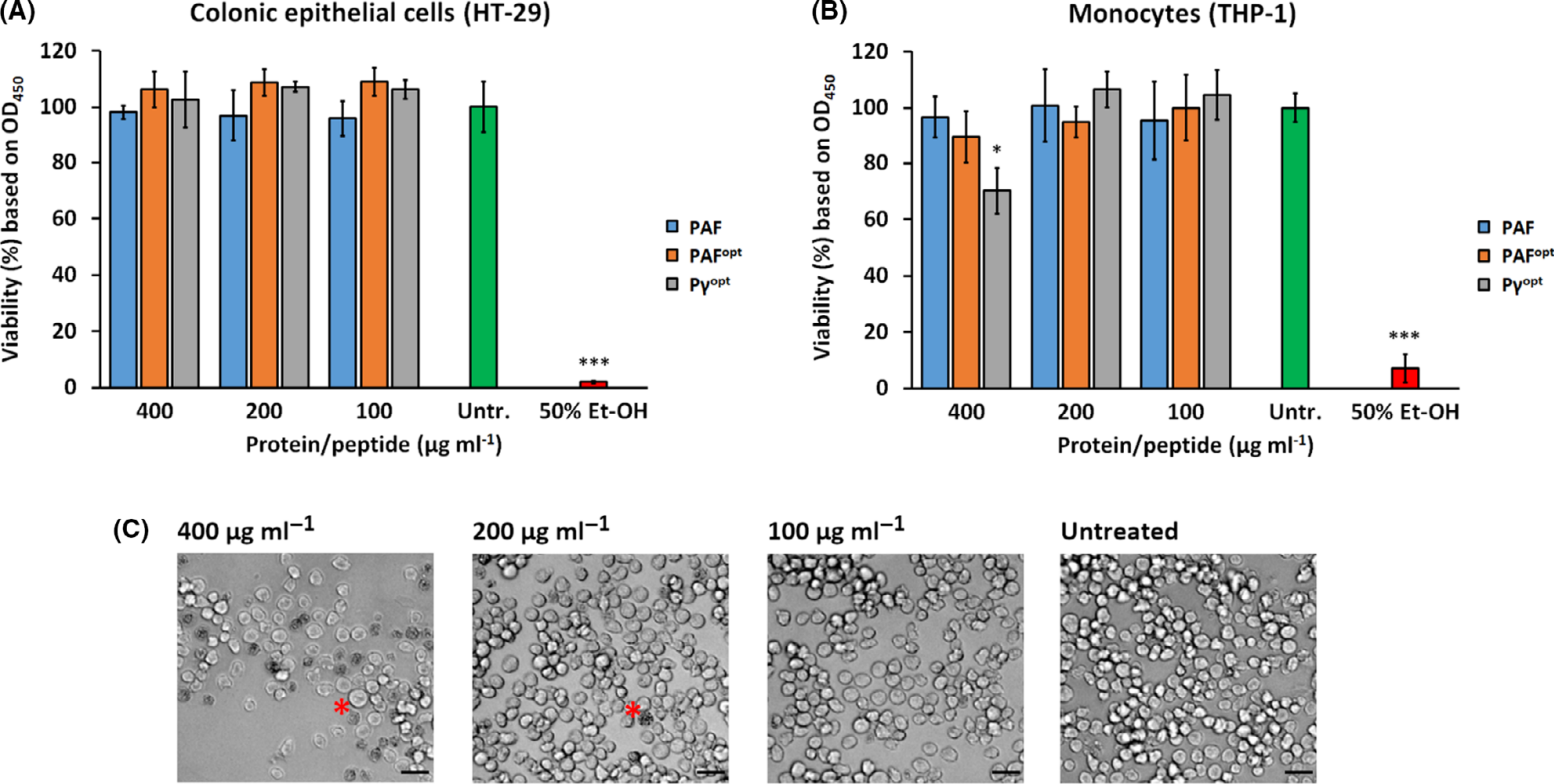
Viability of (A) colonic epithelial cells and (B) monocytes in the presence of PAF, PAF^opt^ and Pγ^opt^ in comparison with the untreated (Untr.) and 50% (v/v) Et‐OH‐treated controls. (C) Visualization of the cytotoxic effect of Pγ^opt^ on monocytes by light microscopy. Red asterisks indicate representatives of a dead cells. Scale bars = 20 µm. Significant differences in (A), and (B) are indicated with *(*P* < 0.05), and ***(*P* < 0.0001) in comparison with the untreated control sample.

*In vitro* cytotoxicity assays with human cell lines are fast and appropriate to detect in advance any harmful effect of a biofungicide candidate molecule before testing it in an *in vivo* animal model system. To prove the safe applicability of PAF, PAF^opt^ and Pγ^opt^ as biofungicides it is essential to exclude their cytotoxic potential on human cell lines that can be inflicted by direct contact with these molecules. In our previous study, we could show that these APs had no adverse effects on keratinocytes and fibroblasts, two major cell types in the epidermal and dermal layer of the skin (Sonderegger *et al.*, 2018). Here, we tested their potential toxicity on colonic epithelial cells, which play an important role in nutrient absorption, and in innate and adaptive mucosal immunity; furthermore, on monocytes involved in the human body’s defence against infectious organisms and foreign substances. The data obtained with the CCK8 cell viability test excluded any toxic effects of PAF and PAF^opt^ on colonic epithelial cells (Fig. 1A), and monocytes *in vitro* (Fig. 1B), even when the proteins were applied at concentrations as high as 400 µg ml^‐1^. In contrast, Pγ^opt^ significantly decreased the viability of monocytes at 400 µg ml^−1^ (Fig. 1B), whereas colonic epithelial cells remained unaffected (Fig. 1A). Microscopic analysis of monocytes treated with 200–400 µg ml^−1^ Pγ^opt^ revealed the presence of abundant dead cells in comparison with the treatment with lower peptide concentrations or to the untreated control (Fig. 1C).

### Effect of PAF, PAF^opt^ and Pγ^opt^ on plant seedlings

**Fig. 2:**
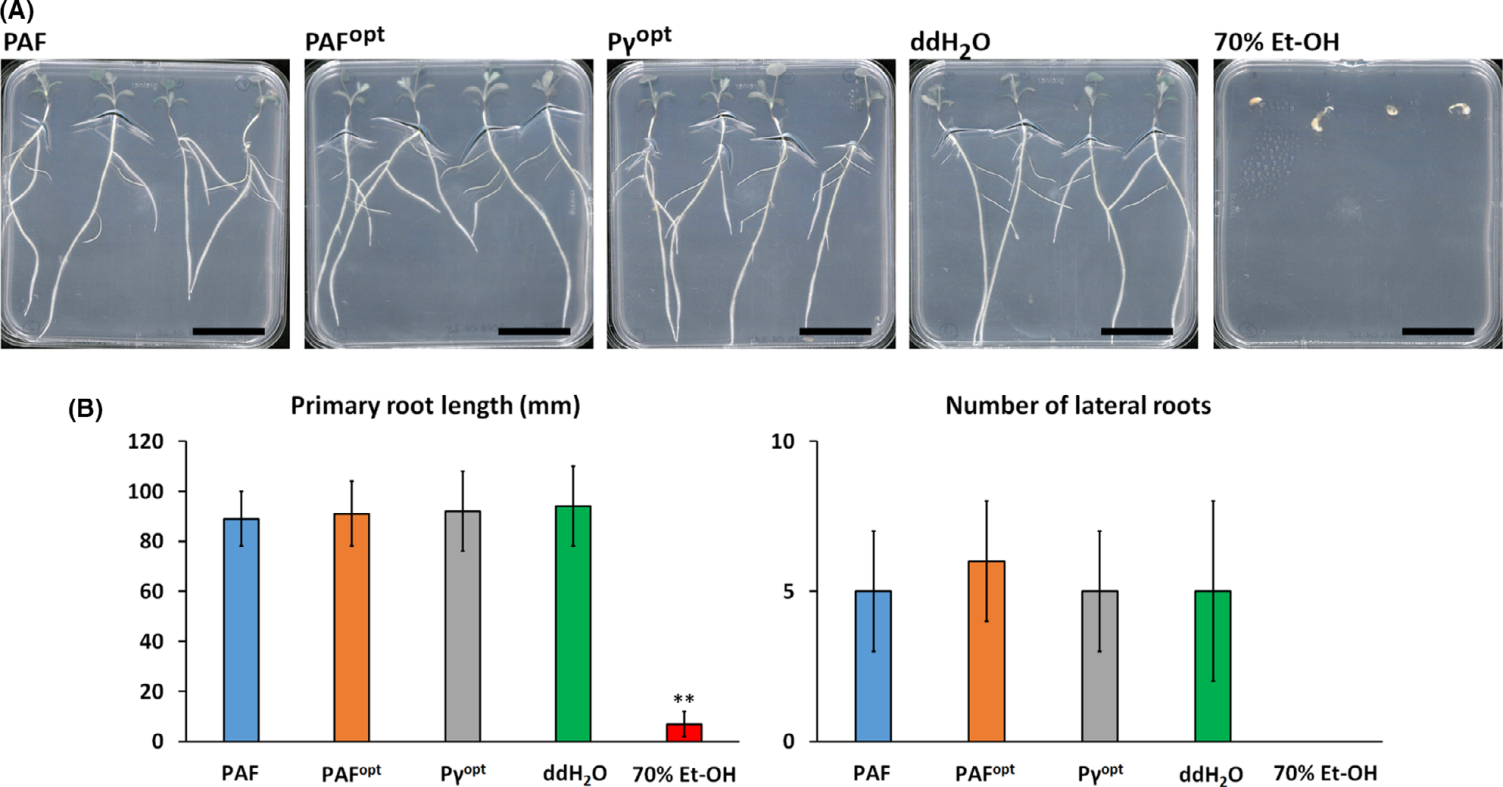
(A) Phenotype of *Medicago truncatula* A‐17 plants grown from seedlings and (B) the length of evolved primary roots and the number of lateral roots after treatment with 400 µg ml^−1^ PAF, PAF^opt^ and Pγ^opt^ for 10 days at 23°C, 60% humidity under continuous illumination (1200 lux) in comparison with ddH_2_O‐ and 70% (v/v) Et‐OH‐treated controls. Scale bars = 30 mm. Significant difference in (B) is indicated with **(*P* < 0.005) in comparison with the ddH_2_O‐treated sample.

*Medicago truncatula* is a small and fast‐growing legume that is easily cultivable on water agar in Petri dishes, thus allowing the reliable investigation of potential toxic effects of pesticides such as APs on intact plants (Barker *et al.*, 2006). For application of PAF, PAF^opt^ and Pγ^opt^ as biocontrol agents in agriculture it is mandatory that they are not harmful to plant seedlings and do not cause any retardation in the plant growth. These effects were investigated on the legume *M. truncatula* A‐17 seedlings by daily treatment of the apical root region with 400 µg ml^−1^ of these APs for 10 days. After the incubation period, no harmful effects were observed. The seedlings grew to healthy mature plants (Fig. 2A) without showing any significant differences in primary root length or number of lateral roots compared with the untreated controls (Fig. 2B).

### Potential of PAF, PAF^opt^ and Pγ^opt^ in plant protection

**Fig. 3:**
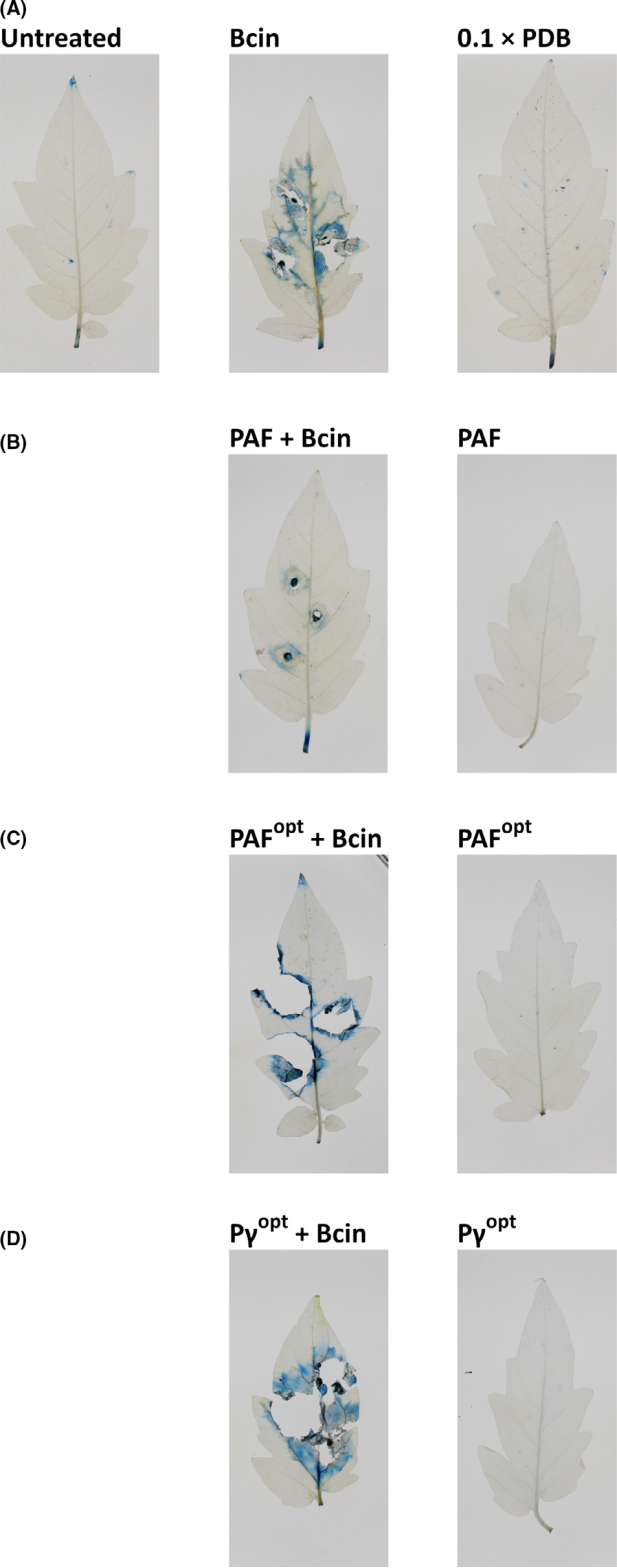
Evan's blue staining of tomato leaves treated with (from left to right) (A) untreated control, *B. cinerea* (Bcin), 0.1 × PDB; (B) *B. cinerea* + PAF (PAF + Bcin), PAF; (C) *B. cinerea* + PAF^opt^ (PAF^opt^ + Bcin), PAF^opt^; (D) *B. cinerea* + Pγ^opt^ (Pγ^opt^ + Bcin), Pγ^opt^. Leaves were kept at 23°C, 60% humidity, and under 12–12 h photoperiodic day‐night simulation at 1200 lux for 4 days. The applied concentration of PAF, PAF^opt^ and Pγ^opt^ was 400 µg ml^−1^. Blue coloured zones or necrotic lesions on the leaves indicate cell death at site of the treatment points with *B. cinerea*.

*Botrytis cinerea* is known as fungal necrotroph of tomato plant leaf tissue (Nambeesan *et al.*, 2012). Considering the promising results from the *in vitro* susceptibility and toxicity tests, the plant protection ability of PAF, PAF^opt^ and Pγ^opt^ was tested against *B. cinerea* infection of tomato plant leaves. To reveal the potential toxic effect of the APs, uninfected leaves were first treated with PAF, PAF^opt^ and Pγ^opt^. A reliable cell viability assay applying Evan’s blue staining (Vijayaraghavareddy *et al.*, 2017) was used to monitor the size of the necrotic zones after treatment. This dye can stain only those cells blue around the treatment site, which have a compromised plasma membrane due to a microbial infection or suffer from membrane disruption by the activity of APs. The PAF, PAF^opt^ and Pγ^opt^ treatment was not toxic to the plants because cell death was not indicated by Evan’s blue staining (PAF, PAF^opt^ and Pγ^opt^ in Fig. 3B, C and D respectively). The same was true for the 0.1 × PDB‐treated control (0.1 × PDB in Fig. 3A). The *B. cinerea* infected but untreated leaves exhibited extensive necrotic lesions and blue coloured zones around the infection points indicating cell death in the consequence of an established and extensive fungal infection (Bcin in Fig. 3A). Next, the tomato leaves were infected with *B. cinerea* and treated with APs. The lack of intensive blue coloured zones and necrotic lesions around the inoculation points indicated that PAF protected tomato plant leaves against *B. cinerea* infection and the invasion of the fungus into the leaf tissue (PAF + Bcin in Fig. 3B). In contrast, PAF^opt^ and Pγ^opt^ was not able to impede fungal infection and necrotic lesions and blue coloured zones appeared at the inoculation points of *B. cinerea* (PAF^opt^ + Bcin and Pγ^opt^ + Bcin in Fig. 3C and D, respectively).

### Protease, thermal and pH tolerance of PAF, PAF^opt^ and Pγ^opt^

The environmental and safe applicability of PAF, PAF^opt^ and Pγ^opt^ as plant protective agents was further evidenced by investigating their tolerance against proteolytic degradation. The proteinase K, a broad‐specific serine protease active within a broad pH and temperature range, and the endopeptidase pepsin, the main digestive enzyme produced in the human stomach and effective at highly acidic pH, were applied in solution digestion experiments to test the potential proteolytic stability of PAF, PAF^opt^ and Pγ^opt^ in the environment and the human digestion system, respectively. All of these APs proved to be highly sensitive to pepsin at pH 2. Intact PAF^opt^ and Pγ^opt^ were not detectable in the solution after two hours of digestion, instead some of their characteristic peptide derivatives appeared (Table S2). Only, a small portion (~9%) of intact PAF was observed at this time, which was finally also degraded by pepsin with prolonged incubation (24 h). In contrast, PAF and Pγ^opt^ proved to be highly resistant against proteinase K: apart from the detectable specific peptide fragments resulting from their degradation (Table S2), ~ 80% of PAF and ~ 39% or Pγ^opt^ were still present in their full‐length form after two hours of enzymatic treatment. However, these completely disappeared after 24 h (Table S2). Almost all the amount of PAF^opt^ was degraded in the presence of proteinase K after two hours, only ~ 1% of the intact protein was detectable among the protein fragments (Table S2). These data indicate that PAF, PAF^opt^ and Pγ^opt^ can be easily degraded by the human digestive system; but PAF and Pγ^opt^ are quite stable against protease degradation under environmental condition.

**Fig. 4:**
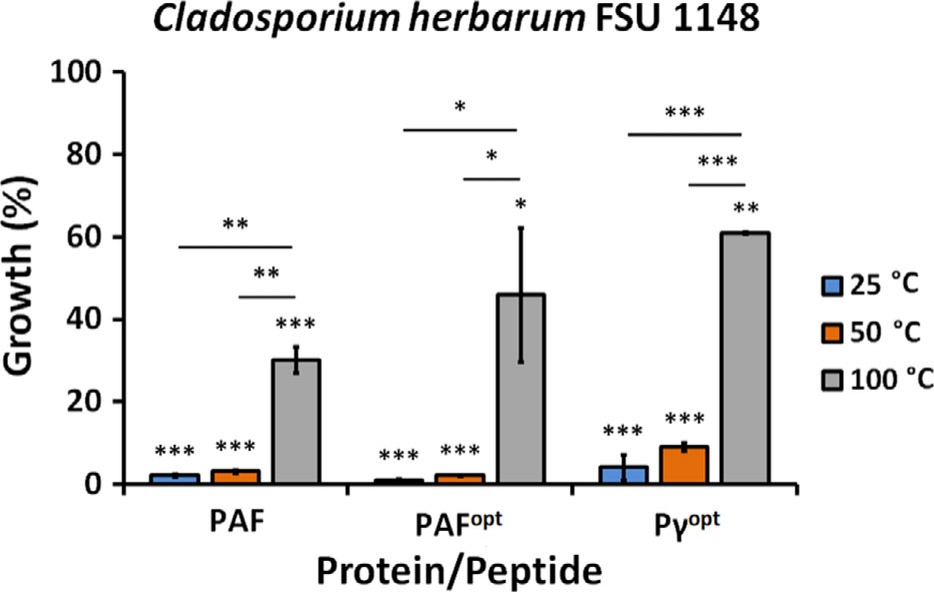
Antifungal activity of PAF, PAF^opt^ and Pγ^opt^ against *C. herbarum* FSU 1148 applied at their respective MIC (Table 2) in broth microdilution test after heat treatment at different temperatures for 60 min. The untreated control culture was referred to 100% growth. Significant differences (*P*‐values) between the growth percentages were determined based on the comparison with the untreated control. Lines indicate statistical comparison between data (growth %) obtained with different treatments. Significant differences are indicated with *(*P* < 0.05), **(*P* < 0.005) and ***(*P* < 0.0001).

PAF, PAF^opt^ and Pγ^opt^ maintained their antifungal activity after heat treatment at 50°C, and their ability to inhibit the growth of *C. herbarum* FSU 1148 was not significantly decreased in comparison with the respective samples treated at 25°C (Fig. 4). Exposure to 100°C caused a significant reduction in the antifungal efficacy of PAF, PAF^opt^ and Pγ^opt^, but all APs retained antifungal activity and reduced the growth of *C. herbarum* FSU 1148 by 70 ± 3.2%, 54 ± 16.3%, 39 ± 0.3%, respectively, in comparison with the untreated growth control (Fig. 4). PAF, PAF^opt^, and Pγ^opt^ maintained their antifungal activity within pH 6–8 without any significant loss of efficacy (data not shown).

## Discussion

In spite of the intensive *in vitro* and *in vivo* laboratory studies for potential use in the fields, only few antimicrobial peptides/proteins have been introduced to the market as a biofungicide product so far (Yan *et al.*, 2015). The commercial development of peptide/protein‐based biofungicides still suffers from several limitations, such as high cost of production, narrow antimicrobial spectrum, susceptibility to proteolytic degradation and toxicity on mammalian or plant cells (Jung and Kang, 2014).

The applied *P. chrysogenum*‐based expression system offers a feasible solution for the commercial production of cysteine‐rich protein‐based biofungicides, reaching yields in the range of mg per litre culture broth of pure protein (Sonderegger *et al.*, 2016, 2018) by a generally recognized as safe (GRAS) status producer (Bourdichon *et al.*, 2012). For example, 2 mg l^−1^ could be achieved for PAF^opt^ (unpublished data), whereas for PAF, concentrations up to 80 mg l^−1^ were reached (Sonderegger *et al.*, 2016).

In this study, we provide for the first time, information about the impact of the modulation of the γ‐core motif of PAF on its antifungal spectrum and efficacy against plant pathogenic filamentous fungi that are important in agriculture (Table 2). Our results emphasize the potential of the evolutionary conserved γ‐core motif for rational AP design to improve the efficacy and modulate the antifungal spectrum. PAF has already been suggested to mitigate the symptoms of barley powdery mildew and wheat leaf rust infections in a concentration‐dependent way on intact plants (Barna *et al.*, 2008). This observation prompted us to further study the efficacy and potential of PAF and PAF^opt^ as biocontrol agents. PAF was able to inhibit *B. cinerea* infection development in tomato plant leaves (Fig. 3B), while PAF^opt^ proved to be ineffective in our plant protection experiments (Fig. 3C); however, both APs inhibited the fungal growth in *in vitro* susceptibility tests (Table 2). One reason for this diverging observation could be that the experimental conditions of *in vitro* tests, for example microdilution assay, differ from the conditions present in other experimental setups which are performed to investigate the protein applicability, such as the plant protection assay. The most possible explanation is that the applied amount of conidia was higher with one magnitude in the plant protection experiments than in the *in vitro* susceptibility test. Presumably, PAF^opt^ could be able to protect the plant against the infectivity of less conidia than applied, or it could be effective on the leaf surface against plant pathogenic fungi other than *B. cinerea*.

The potential effective and safe agricultural application of PAF as a biofungicide and biopreservative agent is further supported by its high tolerance against proteolytic degradation under environmental conditions, and high sensitivity at acidic pH to a digestive enzyme produced in the human stomach (Table S2). Instead, the γ‐core modulated PAF^opt^ proved to be highly sensitive under both conditions (Table S2). ECD spectroscopy revealed a more flexible secondary structure compared with that of the wild‐type PAF (Sonderegger *et al.*, 2018), which could be the reason for an increased accessibility to proteolytic degradation of this PAF variant. The impact of the amino acid exchanges in the γ‐core on the structure of PAF^opt^ will be subject nuclear magnetic resonance analysis in the future.

In agreement with our previous reports on PAF (Batta *et al.*, 2009) and the PAF‐related antifungal protein NFAP from *Neosartorya* (*Aspergillus*) *fischeri* (Galgóczy *et al.*, 2017), also in this study PAF and PAF^opt^ proved to be thermotolerant, retaining fungal growth inhibitory potential after heat treatment (Fig. 4). The decrease in antifungal activity of heat‐treated PAF (Batta *et al.*, 2009) and NFAP (Galgóczy *et al.*, 2017) has been attributed to the loss of the secondary and/or tertiary protein structures. This is noteworthy, as we observed recently that PAF slowly adopts its original secondary structure after thermal unfolding, although it cannot be excluded that a rearrangement of the disulphide bonding occurs in a portion of PAF during this thermal unfolding and refolding process, which results in a decreased antifungal activity (Batta *et al.*, 2009). In contrast, PAF^opt^ seems not to refold, even after four weeks of thermal annealing (Sonderegger *et al.*, 2018). Our data further underline that the physicochemical properties of the γ‐core in PAF^opt^ exert a major role in the antifungal function, whereas the structural flexibility seems of less importance (Sonderegger *et al.*, 2018).

Apart from the full‐length APs, short synthetic peptides spanning the γ‐core motif and their rational designed variants have strong potential as antifungal compounds (Sagaram *et al.*, 2011; Garrigues *et al.*, 2017; Sonderegger *et al.*, 2018). Considering that peptide synthesis is becoming more economic nowadays (Behrendt *et al.*, 2016), the industrial‐scale production of biofungicide peptides is feasible in the near future. In contrast to our previous susceptibility tests with Pγ and Pγ^opt^ against *C. albicans* (Sonderegger *et al.*, 2018), only the Pγ^opt^ (Table 1) was active against plant pathogenic filamentous fungi and proved to be even more potent in some cases than the full‐length PAF or PAF^opt^ (Table 2). In spite of this promising high *in vitro* antifungal activity, sensitivity to proteolytic digestion (Table S2) and the potential cytotoxicity on human cells (Fig. 1C) and inefficiency on plant leaves (Fig. 3D) question its effective and safe applicability as biofungicide or crop preservative. Similar features also limit the use of several other antimicrobial peptides from different origin, which show membrane activity (Li *et al.*, 2017). However, it has to be noted that the applied Pγ^opt^ concentration eliciting cytotoxic effects in human cell lines was much higher than its MIC determined (Table 2). The application of Pγ^opt^ at lower, but still effective concentrations may be well‐tolerated by the host without causing severe side‐effects. Uncovering the molecular basis for the instability and cytotoxic mode of action of Pγ^opt^ in future studies will contribute to refine the rational design approach and the peptide formulation to overcome these obstacles (Hollmann *et al.*, 2018).

Based on our study, we conclude that PAF and PAF^opt^ hold promise as biofungicides that are safely applicable in the fields and as crop preservatives under storage conditions, because these APs are stable and well tolerated by seedlings (Fig. 2), plant leaves (Fig. 3B and C), and human cells (Fig. 1). Our results pave the way for the design and fast development of various APs differing in species specificity and antifungal efficacy. A combinatorial application with other effective APs and/or biofungicides might be promising as well to broaden the antifungal spectrum and further increase the treatment efficacy in the fields. The final prove of concept, however, necessitates field experiments in the future.

## Experimental procedures

### Strains, cell lines and media

The antifungal activity of PAF, PAF^opt^ and their derived γ‐core peptides (Pγ and Pγ^opt^; Table 1) was investigated against 14 potential plant pathogenic filamentous ascomycete isolates listed in the Table 2. These strains were maintained on potato dextrose agar (PDA, Sigma‐Aldrich, St Louis, MO, USA) slants at 4°C, and the susceptibility tests were performed in ten‐fold diluted potato dextrose broth (0.1 × PDB, Sigma‐Aldrich, St Louis, MO, USA). The cytotoxic effect of PAF, PAF^opt^ and Pγ^opt^ was tested on human THP‐1 monocyte cells, and HT‐29 colonic epithelial cells maintained in RPMI‐1640 (no HEPES, phenol red; Gibco, Thermo Fisher Scientific, Waltham, MA, USA) medium supplemented with 10% (v/v) foetal bovine serum (FBS; Gibco Thermo Fisher Scientific, Waltham, MA, USA) and 1% (v/v) antibiotic/antimycotic solution containing 10 000 U ml^−1^ of penicillin, 10 000 µg ml^−1^ of streptomycin and 25 µg ml^−1^ of Amphotericin B (Gibco, Thermo Fisher Scientific, Waltham, MA, USA). Cells were cultured at 37°C in an atmosphere of 5% (v/v) CO_2_ in air.

### Protein production and peptide synthesis

Recombinant PAF and PAF^opt^ were produced in a *P. chrysogenum*‐based expression system and purified according to Sonderegger *et al. *(2016). Pγ and Pγ^opt^ were synthesized on solid phase applying fluorenylmethyloxycarbonyl chemistry as described previously (Sonderegger *et al.*, 2018).

### In vitro antifungal susceptibility test

*In vitro* antifungal susceptibility tests were performed as described previously (Tóth *et al.*, 2016). Briefly, 100 µl of PAF, PAF^opt^, Pγ, or Pγ^opt^ (0.39–800 µg ml^−1^ in twofold dilutions in 0.1 × PDB) was mixed with 100 µl of 2 × 10^5^ conidia ml^−1^ in 0.1 × PDB in flat‐bottom 96‐well microtiter plates (TC Plate 96 Well, Suspension, F; Sarstedt, Nümbrecht, Germany). The plates were incubated for 72 h at 25°C without shaking, and then the absorbance (OD_620_) was measured in well‐scanning mode after shaking the plates for 5 s in a microtiter plate reader (SPECTROstar Nano, BMG Labtech, Ortenberg, Germany). Fresh medium (200 µl 0.1 × PDB) was used for background calibration. The MIC was defined as the lowest antifungal protein or peptide concentration at which growth was not detected (growth ≤ 5%) after 72 h of incubation on the basis of the OD_620_ values as compared to the untreated control (100 µl 0.1 × PDB was mixed with 100 µl of 10^5^ conidia ml^−1^ in 0.1 × PDB). The growth ability of *F. oxysporum* SZMC 6237J in the presence of PAF, PAF^opt^ or Pγ^opt^ was also calculated in comparison with the untreated control and was given in percentage. The absorbance of the untreated control culture was referred to 100% growth for the calculations. Susceptibility tests were repeated at least two times including three technical replicates.

### Cell viability tests on human cells lines

CCK8 cell proliferation and cytotoxicity assay kit (Dojindo Molecular Technologies Inc.; Rockville, MD, USA) was applied to reveal the possible toxic effect of PAF, PAF^opt^ or Pγ^opt^ on human cell lines. Cell viability tests were performed according the manufacturer’s instruction with slight modifications. Cells (20 000 cells in a well) were preincubated in a flat‐bottom 96‐well microtiter plate (TC Plate 96 Well, Standard, F; Nümbrecht, Germany) in 100 µl (in the case of HT‐29) or 80 µl (in the case of THP‐1) RPMI‐1640 medium without phenol red (Gibco, Thermo Fisher Scientific, Waltham, MA, USA) for 24 h in a humidified incubator at 37°C in an atmosphere of 5% (v/v) CO_2_ in air. For the experiment, the medium was then replaced by 100 µl of fresh medium supplemented with 100–400 µg ml^−1^ PAF, PAF^opt^ or Pγ^opt^ in twofold dilution on the adherent HT‐29 colonic epithelial cells; while the preincubated non‐adherent THP‐1 monocyte cell cultures was supplemented with 20 µl PAF, PAF^opt^ or Pγ^opt^ solutions in RPMI‐1640 to reach the 100–400 µg ml^−1^ final concentration (twofold dilution) in 100 µl volume. Medium without AP supplementation was used for the controls. After 24 h of incubation, medium was replaced to 100 µl AP‐free RPMI‐1640 (without phenol red) on HT‐29 cells. Ten microlitre of the CCK‐8 solution was mixed to each well of HT‐29 and THP‐1 cell cultures by gently pipetting up and down. Absorbance was measured at 450 nm using a microplate reader (Hidex Sense Microplate Reader, Turku, Finland) after 2 h (colonic epithelial cells) or 4 h (monocytes) of incubation. Cells treated with 50% (v/v) ethanol for 10 min were used as dead control. For calculation of the cell viability in the presence of PAF, PAF^opt^, Pγ^opt^ or 50% (v/v) ethanol, the absorbance of the untreated control cultures (100 µl RPMI‐1640 or DMEM without phenol red) were set to be 100% growth. Fresh medium without phenol red (100 µl) was used for background calibration.

### Toxicity tests on M. truncatula seedling

For the toxicity tests, *M. truncatula* A‐17 seeds were treated with 96% (v/v) sulphuric acid for 5 min, then with 0.1% (w/v) mercuric chloride solution for 3 min at room temperature. After each treatment, seeds were washed with cold ddH_2_O three times. Treated seeds were plated on 1% (w/v) water agar (Agar HP 696; Kalys, Bernin, France) to allow them to germinate for three days at 4°C. Four seedlings with 3–4 mm root in length were transferred in a lane (keeping a 20 mm distance from the top) to a square Petri dish (120 × 120 × 17 mm Bio‐One Square Petri Dishes with Vents; Greiner, Sigma‐Aldrich, St Louis, MO, USA) containing fresh 1% (w/v) water agar (Agar HP 696; Kalys, Bernin, France). The apical region of the evolved primary root was treated for 10 days with daily dropping 20 µl aqueous solution of 400 µg ml^−1^ PAF, PAF^opt^ or Pγ^opt^. Plates were incubated in a humid (60%) plant growth chamber at 23°C under continuous illumination (1200 lux) of the leaf region. The root region was kept in dark covering this part of the square Petri dish with aluminium foil from 20 mm distance from the top. The primary root length was measured (in mm) and the number of lateral roots were counted on day 10 of the incubation. ddH_2_O‐ and 70% (v/v) ethanol‐treated seedlings were used as growth and dead control, respectively. Toxicity tests were repeated at least two times, and twelve seedlings were involved in each treatment.

### Plant protection experiments

Seeds of tomato plants (*Solanum lycopersicum* L. cv. Ailsa Craig) were germinated for 3 days at 27°C under darkness. Tomato seedlings were transferred to Perlite for 14 days and were grown in a controlled environment under 200 μmol m^−2^ s^−1^ photon flux density with 12/12‐h light/dark period, a day/night temperatures of 23/20°C and a relative humidity of 55–60% for 4 weeks in hydroponic culture (Poór *et al.*, 2011). The experiments were conducted from 9 a.m. and were repeated three times.

For the plant protection assay, we adopted the pathogenicity test method described by El Oirdi *et al. *(2010, 2011) with slight modifications. Detached tomato plant leaves were laid on Petri dishes containing three sterilized filter papers (0113A00009 qualitative filter paper; Filters Fioroni, Ingré, France) wetted with sterile ddH_2_O. (i) For the infection control, 10 µl *B. cinerea* SZMC 21472 conidial suspension (1 × 10^7^ conidia ml^−1^), (ii) for the AP toxicity testing 10 µl of 400 µg ml^−1^ PAF, PAF^opt^ or Pγ^opt^ (iii) for the plant protection investigation 10 µl *B. cinerea* conidial suspension (1 × 10^7^ conidia ml^−1^) containing 400 µg ml^−1^ PAF, PAF^opt^ or Pγ^opt^, and (iv) for the uninfected control 10 µl 0.1 × PDB was dropped onto abaxial leaf epidermis in three points between the later veins and left to dry on the surface at room temperature. Conidial suspension and AP solutions were prepared in 0.1 × PDB for the tests. After these treatments, leaves were kept in a humid (60%) plant growth chamber for four days at 23°C under photoperiodic day‐night simulation (12–12 h with or without illumination at 1200 lux). Leaf without any treatment was used as an untreated control. After the incubation period, leaves were collected and the necrotic zone around the treatment points and necrotic lesions were visualized by Evan’s blue staining. Briefly, the leaves were stained with 1% (w/v) Evan’s blue (Sigma‐Aldrich, St Louis, MO, USA) for 10 min according to Kato *et al. *(2007), then rinsed with distiled water until they were fully decolorized. Then, chlorophyll content was eliminated by boiling in 96% (v/v) ethanol for 15 min. Leaves were stored into glycerine:water:alcohol (4:4:2) solution and photographed by Canon EOS 700D camera (Tokyo, Japan). Three leaves for each treatment were used in one experiment. Plant protection experiments were repeated twice.

### Protein and peptide stability investigations

Resistance of PAF, PAF^opt^ and Pγ^opt^ against proteolytic enzymes such as pepsin and proteinase K (Sigma‐Aldrich, St Louis, MO, USA) was investigated by in solution digestion and liquid chromatography‐electrospray ionization‐tandem mass spectrometry analysis of the digested products. For this, 20 µl protein/peptide solution (1 µg ml^−1^) was mixed with a buffer containing 25 mM ammonium bicarbonate (pH = 8.0) for proteinase K digestion, or in H_2_O containing 0.1% (v/v) formic acid (pH = 2.0) for pepsin digestion. Reaction mixtures were incubated at 25°C (proteinase K) and 37°C (pepsin) for 2 and 24 h. Enzyme peptide mass ratio was 1:20. Digested samples were analysed on a Waters NanoAcquity UPLC (Waters MS Technologies, Manchester, UK) system coupled with a Q Exactive Quadrupole‐Orbitrap mass spectrometer (Thermo Fisher Scientific, Waltham, MA, USA). Liquid chromatography conditions were the followings: flow rate: 350 nl min^−1^; eluent A: water with 0.1% (v/v) formic acid, eluent B: acetonitrile with 0.1%(v/v) formic acid; gradient: 40 min, 3–40% (v/v) B eluent; column: Waters BEH130 C18 75 lm/250 mm column with 1.7 μm particle size C18 packing (Waters Inc., Milford, MA, USA). The presence and ratio of full‐length PAF, PAF^opt^ and Pγ^opt^ and their characteristic peptide fragments in the solutions after digestion were detected by peptide mass mapping (Protein Prospector, MS‐fit; http://prospector.ucsf.edu/).

To investigate the pH and temperature tolerance of PAF, PAF^opt^ and Pγ^opt^, the antifungal susceptibility test was repeated against *C. herbarum* FSU 1148 at the previously detected MIC concentration of these APs, but the 0.1 × PDB was prepared in sodium‐phosphate buffer (50 mM, pH 6.0–8.0), or the protein/peptide solutions were exposed to different temperatures (25, 50, 100°C) for 60 min. Respective fresh media were used for background calibration. For calculation of the growth ability the absorbance of the untreated control cultures (medium without PAF, PAF^opt^ or Pγ^opt^) were referred to 100% growth. These susceptibility tests were prepared in duplicates and repeated three times.

### Statistical analyses

Microsoft Excel 2016 software (Microsoft, Edmond, WA, USA) was used to calculate standard deviations and to determine the significance values (two sample *t*‐test). Significance was defined as *P* < 0.05, based on the followings: *P ≤ 0.05, **P ≤ 0.005 and ***P ≤ 0.0001.

## Conflict of interest

None declared.

## Supporting information


**Table S1.** Growth percentages (%) of *Fusarium oxysporum* SZMC 6237J in the presence of different concentrations of PAF, PAF^opt^, and Pγ^opt^ after incubation for 72 h at 25°C in 0.1 × PDB.

**Table S2.** Identified peptide fragments of pepsin of proteinase K digested PAF, PAF^opt^, Pγ^opt^ and their intensity after 2 or 24 h of proteolytic enzyme treatment.
