# Supplementary material for: The potential use of the Penicillium chrysogenum antifungal protein PAF, the designed variant PAFopt and its γ‐core peptide Pγopt in plant protection
Source: Microb Biotechnol. 2020 Mar 24;13(5):1403–14. doi: 10.1111/1751-7915.13559 (PMC7415367; doi:10.1111/1751-7915.13559)
Supplement: Supplementary file 1 — Table S1. Growth percentages (%) of Fusarium oxysporum SZMC 6237J in the presence of different concentrations of PAF, PAFopt, and Pγopt after incubation for 72 h at 25°C in 0.1 × PDB. [file MBT2-13-1403-s001.docx]

***Supporting Information***

**The potential use of the *Penicillium chrysogenum* antifungal protein PAF, the designed variant PAF^opt^ and its γ-core peptide Pγ^opt^ in plant protection**

**Liliána Tóth,^1^ Éva Boros,^2^ Péter Poór,^3^ Attila Ördög,^3^ Zoltán Kele,^4^ Györgyi Váradi,^4^ Jeanett Holzknecht,^5^ Doris Bratschun-Khan,^5^ István Nagy,^2^ Gábor K. Tóth,^4,6^ Gábor Rákhely,^7,8^ Florentine Marx,^5,*^ László Galgóczy^1,7,*^**

^1^ *Institute of Plant Biology, Biological Research Centre, Temesvári krt. 62, H-6726 Szeged, Hungary*

^2^ *Institute of Biochemistry, Biological Research Centre, Temesvári krt. 62, H-6726 Szeged, Hungary*

^3^ *Department of Plant Biology, Faculty of Science and Informatics, University of Szeged, Közép fasor 52, H-6726, Szeged, Hungary*

^4^ *Department of Medical Chemistry, Faculty of Medicine, University of Szeged, Dóm tér 8, H-6720 Szeged, Hungary*

^5^ *Institute of Molecular Biology, Biocenter, Medical University of Innsbruck, Innrain 80-82, A-6020 Innsbruck, Austria*

^6^ *MTA-SZTE Biomimetic Systems Research Group, University of Szeged, Dóm tér 8, H-6720 Szeged, Hungary*

^7^ *Department of Biotechnology, Faculty of Science and Informatics, University of Szeged, Közép fasor 52, H-6726, Szeged, Hungary*

^8^ *Institute of Biophysics, Biological Research Centre, Temesvári krt. 62, H-6726 Szeged, Hungary*

*For correspondence. E-mail florentine.marx@i-med.ac.at; Tel. +43 512 9003 70207; Fax +43/(0)512 9003 73100 (FM). E-mail galgoczi@bio.u-szeged.hu; Tel. +36 62 546 936; Fax +36 62 544 352 (LG).

**Table S1.** Growth percentages (%) of *Fusarium oxysporum* SZMC 6237J in the presence of different concentrations of PAF, PAF^opt^, and Pγ^opt^ after incubation for 72 hours at 25 °C in 0.1 × PDB.

| ***Fusarium oxysporum* SZMC 6237J** | | | | | | | | | |
| --- | --- | --- | --- | --- | --- | --- | --- | --- | --- |
| **Cc. (µg ml^-1^) /**  **Protein or peptide** | **400** | **200** | **100** | **50** | **25** | **12.5** | **6.25** | **3.125** | **0** |
| **PAF** | **5±2.2** | 36±3.4 | 60±4.2 | 77±2.8 | 96±9.3 | 95±7.6 | 108±9.3 | 115±5.2 | 100±3.7 |
| **PAF^opt^** | 87±5.5 | 80±14.8 | **5±1.4** | 16±3.5 | 28±3.7 | 50±11.3 | 88±9.6 | 104±4.2 | 100±4.4 |
| **Pγ^opt^** | 0±0.3 | 0±0.6 | 0±0.2 | 0±0.4 | **0±0.7** | 52±8.1 | 66±8.3 | 96±11.4 | 101±4.2 |

Red colour indicates total growth inhibition (growth < 5%), orange colour reduced growth ability (growth between 5% and 85%), green colour full growth (growth above 85%). The untreated control was defined as 100% of growth. Minimal inhibitory concentrations are indicated in bold letters and with black frame.

**Table S2.** Identified peptide fragments of pepsin of proteinase K digested PAF, PAF^opt^, Pγ^opt^ and their intensity after 2 or 24 hours of proteolytic enzyme treatment.

| **Sequence** | **Intensity** | | | **Approximately ratio (%)**  **0 h : 2 h : 24 h** |
| --- | --- | --- | --- | --- |
|  | **0 hours** | **2 hours** | **24 hours** |  |
| **Pepsin** | | | | |
| **PAF:**  **A**K**Y**TGKCTKSKNECK**Y**KND**A**GKDT**FI**KCPK**F**DNKKCTKDNNKCTVDT**Y**NN**A**VDC**D** | | | | |
| Full length protein | 3828843537 | 357920872 | 0 | 100.00 : 9.35: 0.00 |
| IKCPKF-VDCD | 0 | 820905 | 56804772 | 0.00 : 1.45 : 100.00 |
| **PAF^opt^:**  **A**K**Y**TGKCKTKKNKCK**Y**KND**A**GKDT**FI**KCPK**F**DNKKCTKDNNKCTVDT**Y**NN**A**VDC**D** | | | | |
| Full length protein | 31230833020 | 0 | 0 | 100.00 . 0.00. : 0.00 |
| IKCPKF-VDCD | 0 | 367602 | 479703744 | 0.00 : 0.08 : 100.00 |
| **Pγ^opt^**:  K**Y**TGKCKTKKNKC**K** | | | | |
| Full length peptide | 8609514011 | 541403 | 0 | 100.00 : 0.01 : 0.00 |
| KY | 0 | 144476609 | 3098993582 | 0.00 : 4.66 : 100.00 |
| **Proteinase K** | | | | |
| **PAF:**  **A**K**YT**GKC**T**KSKN**E**CK**Y**KND**A**GKD**TFI**KCPK**F**DNKKC**T**KDNNKC**TV**D**TY**NN**AV**DC**D** | | | | |
| Full length protein | 3828843537 | 3058779489 | 0 | 100.00 : 79.89 : 0.00 |
| IKCPKF-VDCD | 0 | 83807800 | 9314878 | 0.00 : 100.00: 11.12 |
| IKCPKF-DCD | 0 | 12886124 | 10713440 | 0.00 : 100.00: 83.14 |
| KCPKF-DCD | 0 | 0 | 1476230 | 0.00. : 0.00 : 100.00 |
| **PAF^opt^:**  **A**K**YT**GKCK**T**KKNKCK**Y**KND**A**GKD**TFI**KCPK**F**DNKKC**T**KDNNKC**TV**D**TY**NN**AV**DC**D** | | | | |
| Full length protein | 31230833020 | 394652275 | 0 | 100.00 : 1.26 : 0.00 |
| IKCPKF-VDCD | 0 | 111533024 | 8501227 | 0.00 : 100.00 : 7.62 |
| IKCPKF-DCD | 0 | 31206868 | 5400116 | 0.00 : 100.00 : 17.30 |
| KCPKF-DCD | 0 | 0 | 886804 | 0.00 : 0.00: 100.00 |
| **Pγ^opt^**:  K**YT**GKCK**T**KK NK**CK** | | | | |
| Full length peptide | 8609514011 | 3376923628 | 0 | 100.00 : 39.22 : 0.00 |
| KY | 0 | 79029247 | 2044076240 | 0.00 : 3.87 : 100.00 |

Underlined bold letters indicate the cleavage sites (predicted by Protein Prospector MS-Digest: <http://prospector.ucsf.edu/prospector/cgi-bin/msform.cgi?form=msdigest>), while the identified peptide fragments are highlighted in red. For the ratio calculation the highest intensity score of a certain protein or peptide was taken as 100.
