# Supplementary material for: The potential use of the Penicillium chrysogenum antifungal protein PAF, the designed variant PAFopt and its γ‐core peptide Pγopt in plant protection
Source: Microb Biotechnol. 2020 Mar 24;13(5):1403–14. doi: 10.1111/1751-7915.13559 (PMC7415367; doi:10.1111/1751-7915.13559)
Supplement: Supplementary file 2 — Table S2. Identified peptide fragments of pepsin of proteinase K digested PAF, PAFopt, Pγopt and their intensity after 2 or 24 h of proteolytic enzyme treatment. [file MBT2-13-1403-s002.docx]

**Table S1.** Growth percentages (%) of *Fusarium oxysporum* SZMC 6237J in the presence of different concentrations of PAF, PAF^opt^, and Pγ^opt^ after incubation for 72 hours at 25 °C in 0.1 × PDB.

| ***Fusarium oxysporum* SZMC 6237J** | | | | | | | | | |
| --- | --- | --- | --- | --- | --- | --- | --- | --- | --- |
| **Cc. (µg ml^-1^) /**  **Protein or peptide** | **400** | **200** | **100** | **50** | **25** | **12.5** | **6.25** | **3.125** | **0** |
| **PAF** | **5±2.2** | 36±3.4 | 60±4.2 | 77±2.8 | 96±9.3 | 95±7.6 | 108±9.3 | 115±5.2 | 100±3.7 |
| **PAF^opt^** | 87±5.5 | 80±14.8 | **5±1.4** | 16±3.5 | 28±3.7 | 50±11.3 | 88±9.6 | 104±4.2 | 100±4.4 |
| **Pγ^opt^** | 0±0.3 | 0±0.6 | 0±0.2 | 0±0.4 | **0±0.7** | 52±8.1 | 66±8.3 | 96±11.4 | 101±4.2 |

Red colour indicates total growth inhibition (growth < 5%), orange colour reduced growth ability (growth between 5% and 85%), green colour full growth (growth above 85%). The untreated control was defined as 100% of growth. Minimal inhibitory concentrations are indicated in bold letters and with black frame.
